# Improved sample preparation for direct quantitative detection of *Escherichia coli* O157 in soil using qPCR without pre‐enrichment

**DOI:** 10.1111/1751-7915.12737

**Published:** 2017-06-06

**Authors:** Callum J. Highmore, Steve D. Rothwell, Charles W. Keevil

**Affiliations:** ^1^ Environmental Healthcare UnitFaculty of Natural & Environmental SciencesUniversity of SouthamptonSouthamptonUKSO17 1BJ; ^2^ Vitacress Salads LtdLower Link FarmSt Mary BourneAndoverUKSP11 6DB

## Abstract

The prominence of fresh produce as a vehicle for foodborne pathogens such as enterohaemorrhagic *Escherichia coli* (EHEC) O157 is rising, where disease cases can cause hospitalization and in some cases death. This rise emphasises the necessity for accurate and sensitive methods for detection of pathogens in soil, potential sources of contamination of fresh produce. The complexity of the soil matrix has previously proven prohibitive to pathogen detection via molecular methods without the use of a culture enrichment step, thereby excluding the detection of viable but non‐culturable cells. Here, a sample preparation procedure to facilitate a direct qPCR assay is developed for the detection of *E*.* coli* O157 in soil, bypassing culture steps in favour of sample separation through pulsification release and filtration. In sand and peat‐based compost, the method is sensitive to 10 CFU g^−1^ soil. When testing soils from agricultural sites, it was found that several were qPCR positive for *E*.* coli* O157 while being culture‐negative, with peat‐based compost possessing a concentration of 200 *tir* gene copies per gram. This procedure offers a rapid, quantitative assessment of the potential presence of *E*.* coli* O157 in soils which can act as a prescreen of their suitability to grow fresh produce safely.

## Introduction

The increase in popularity of fresh produce has caused a rise in prominence of fresh produce‐associated outbreaks of foodborne disease. Between 2010 and 2013, two outbreaks of *Escherichia coli* O157 in the United Kingdom caused 251 and 19 disease cases, respectively (Pennington, 2014). In both outbreaks, the source was considered to be contaminated growing media, an essential component of food production which poses severe limitations to the detection of bacterial pathogens due to its complex physicochemical matrix (Wilks and Keevil, 2013).

Soil and growing media such as peat‐based compost and coir compost can become contaminated by foodborne pathogens such as *E*.* coli* O157:H7 through the application of animal faeces to agricultural land, both accidentally by roaming animals and overflying birds and deliberately as fertilizer. It has been found that animals such as rats are able to shed the pathogen for up to 11 days, whereas infected pigeons shed for 29 days (Cizek *et al*., 2000). Enterohaemorrhagic *E*.* coli* (EHEC) has been detected in a range of birds, where the *stx2* toxin gene was detected by qPCR in 23% of 412 *E*.* coli* isolates from eight of 15 avian species. The species in which the gene was most prevalent were raven, turkey and pigeon, where isolates also originated in mallard ducks and pheasants (Chandran and Mazumder, 2014).

Manure and slurries are a major source of fertilizer for agricultural land. In Scotland, they comprise 96% of organic waste spread on land (1997a). Several studies have assessed the prevalence of EHEC in cattle, one using traditional culture techniques to examine the presence of *E*.* coli* O157:H7 in 3570 dairy cattle across 60 herds. It was found that 10 cattle were positive for the bacterium (0.28%), across five herds (Hancock *et al*., 1994). Another study compared the prevalence of the pathogen across herds associated with *E*.* coli* O157:H7 outbreaks in humans. Within herds, prevalence ranged from 1.3% to 9.5%, whereas in herds of cattle not associated with any *E*.* coli* O157:H7 cases in humans, it was found that 0–6.1% of cattle were infected with the pathogen (1997b). In addition to contaminating agricultural land, there is evidence that the application of biosolids can increase the population of EHEC in the soil; one study notes an increase in 2.62 orders of magnitude of indicator *E*.* coli* (Unc *et al*., 2006).

Currently, detection of pathogens in growing media is achieved through traditional culture methods (Islam *et al*., 2004; Avery *et al*., 2005; Wadamori *et al*., 2016), where even assays using molecular techniques such as qPCR commonly depend on an enrichment culture step to amplify a signal and dilute out potential inhibitors (Nam *et al*., 2005). However, enrichment culture methods exclude the presence of dead and viable but non‐culturable (VBNC) cells (Colwell and Grimes, 2000; Li *et al*., 2014), both of which are important for determining the safety of agricultural growing media. Dead cells indicate previous contamination of a sample and VBNC cells present in a sample pose a direct threat to the safety of a growing medium. Dinu and Bach have shown that a VBNC state can be induced in *E*.* coli* O157:H7 through exposure to conditions present on the phylloplane of lettuce (Dinu and Bach, 2011), highlighting the potential population of produce‐associated pathogens that could go undetected by currently used culture methods. The pathogenicity of VBNC *E*.* coli* O157:H7 cells has been established, where expression of *stx* toxin genes has been documented despite the stressed state (Liu *et al*., 2010). Resuscitation of VBNC cells is also possible; allowing the establishment of infection provided they could reach an appropriate host. Other pathogens, *Listeria monocytogenes* (Cappelier *et al*., 2007) and *Vibrio alginolyticus* (Du *et al*., 2007), have retained their pathogenicity following resuscitation.

While culture methods may produce false‐negative results in pathogen detection, testing large volumes of growing media through molecular methods is also restricted by the complexity of the matrix. Separation of the bacterial sample is essential due to the presence of PCR inhibitors such as humic acids (Tebbe and Vahjen, 1993). Attachment of bacterial cells to soil particles must be reduced as much as possible prior to sample concentration to effectively utilize molecular detection methods for large samples of soil. The necessity of sample concentration is emphasized by the limitations of most commercial DNA extraction kits, recommending only up to 0.25 g of soil per sample.

To best overcome these problems and understand the risk of *E*.* coli* O157 in soil and growing media, this study employs a cost effective, qPCR assay that removes the need for culture‐based pre‐enrichment. The Pulsifier is used to facilitate bacterial sample separation from the soil matrix through high‐frequency oscillation, degrading the matrix less than a paddle‐based homogenizer (Wu *et al*., 2003). Vacuum filtration is then used to concentrate a large soil sample (25 g) to a volume suitable for DNA extraction by an effective commercial kit (Mo Bio, Carlsbad, CA, USA) (Mahmoudi *et al*., 2011), and the *E*.* coli* O157 population is quantified using qPCR. The assay targets the *E*.* coli* O157 *tir* (translocated intimin receptor) gene, as it is specific to serotype O157 strains. The primer is also available in a commercial kit (PrimerDesign, Chandler's Ford, UK), which allows the procedure to be more easily applied in industrial laboratories. Using this diagnostic assay, it was revealed that several soils of agricultural origin were positive for *E*.* coli* O157 although there was no corresponding culture recovery.

## Results

**Figure 1:**
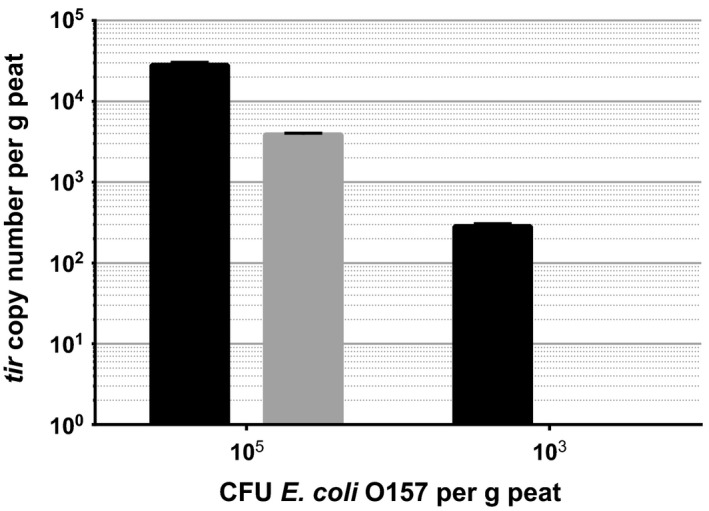
*tir* gene copy number detected in peat‐based compost using the Pulsifier (black) and the Stomacher (grey). Error bars represent SEM. *P* < 0.005.

To optimize separation of bacterial suspension from the complex matrix of soil, the Pulsifier was tested against the better established Stomacher. This sample separation step was followed by filtration, DNA isolation and qPCR, to determine which process is more appropriate for the pathogen detection assay. In peat‐based compost, a greater recovery of the bacterial sample was attained when using the Pulsifier (Fig. 1). In samples inoculated with 10^5^ CFU *E*.* coli* O157 per g, the Stomacher causes a log reduction in detection when compared with the Pulsifier. At an inoculum concentration of 10^3^ CFU *E*.* coli* O157 per g, the Stomacher prevents the detection of any *tir* genes by the qPCR assay.

**Figure 2:**
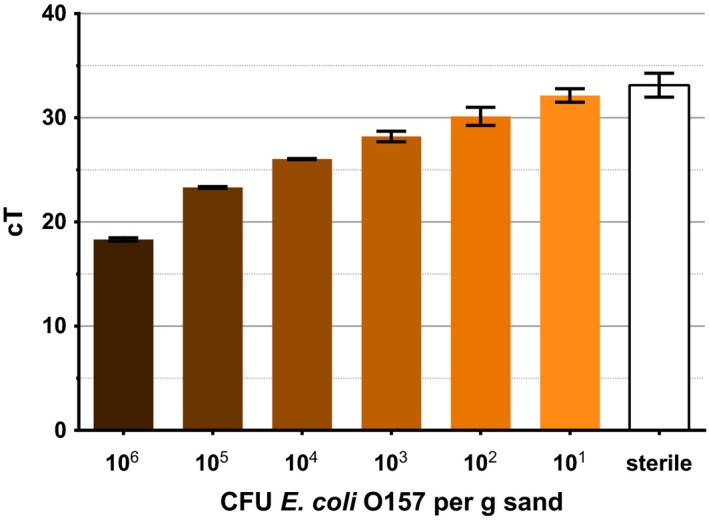
cT values of sand inoculated with different concentrations of *E*.* coli* O157. Sterile indicates sterilized sand not inoculated with bacteria. Error bars indicate SEM. *R* ^2^=0.9756.

**Figure 3:**
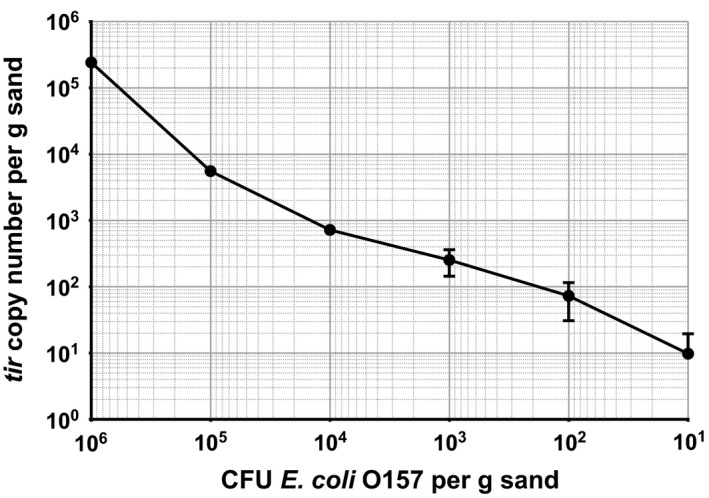
*tir* copy number detected in sand containing a range of concentrations of *E*.* coli* O157. Error bars indicate SEM.

The assay is capable of detecting *E*.* coli* O157 in sterilized sand to a sensitivity of 10 CFU g^−1^ (Fig. 2). The control sample, containing sterile, uninoculated soil, showed gene amplification passing the cycle threshold with an average cT of 33. Following inoculation with *E*.* coli* O157, the sample containing 10^6^ CFU g^−1^ soil passed the cycle threshold at cycle 18 and each successive log dilution of inoculum had a correspondingly greater cT value. Despite this, a log increase in inoculation concentration does not correspond to a log increase in *tir* copies detected (Fig. 3). Using a standard curve, the cT values are converted to *tir* gene copy numbers to quantify bacterial numbers within the soil (Figs 3 and 5). The detected gene copies closely correspond to low inoculum concentrations, 73 *tir* copies were detected per gram of soil at an inoculum of 100 CFU *E*.* coli* O157, and 10 copies were detected when 10 CFU was inoculated (Fig. 3). At higher inoculum concentrations, fewer *tir* gene copies are detected per inoculated CFU (Fig. 3). Despite heat sterilization, an average of two gene copies was found in sterile sand.

**Figure 4:**
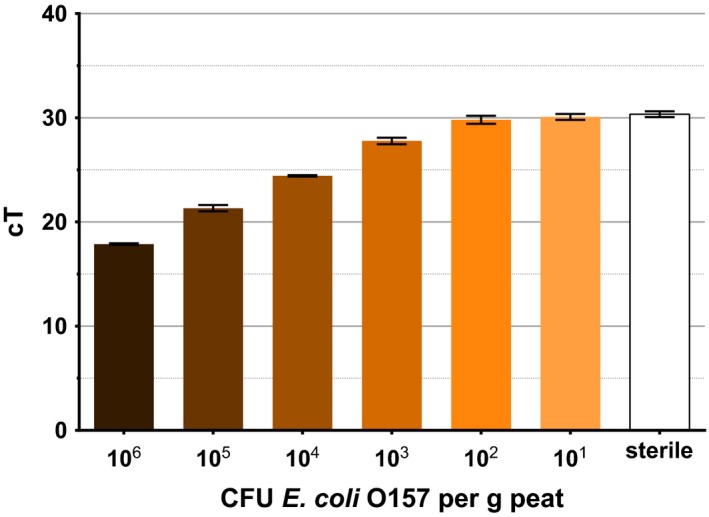
cT values of peat‐based compost inoculated with different concentrations of *E*.* coli* O157. Sterile indicates soil not inoculated with bacteria, heat‐treated and DNase I‐treated. Error bars indicate SEM. *R* ^2^=0.9878.

**Figure 5:**
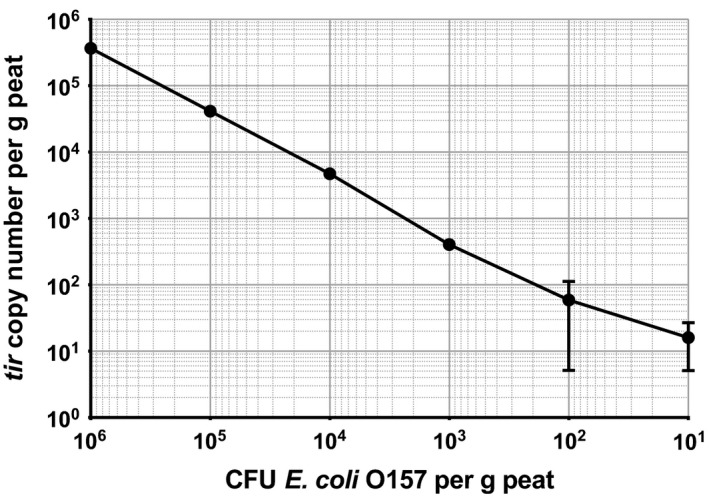
*tir* copy number detected in sand containing a range of concentrations of *E*.* coli* O157. The average copy number generated by the sterile sample was subtracted from the other columns to remove the *tir* gene background. Error bars indicate SEM.

In peat‐based compost, the assay can reach a similar sensitivity of detection. To reduce the quantity of *tir* genes detected in sterilized soil, peat samples were treated with DNase I (Sigma‐Aldrich, St. Louis, MO, USA) before inoculation; however, no further reduction in *tir* gene detection was achieved. The peat sample inoculated with 10^6^ CFU g^−1^ soil *E*.* coli* O157 passed the threshold at cycle 17, and similar to sand, each reduction in inoculum concentration caused an increase in average cT value (Fig. 4). Fifty‐nine gene copies per gram of peat‐based compost were detected in the DNAse I‐treated sample. This average was subtracted from the total detected *tir* genes as a background level of the gene in the compost, so remaining *tir* gene detection could be attributed to the *E*.* coli* O157 inocula. In the absence of the background *tir* gene copies, the sensitivity of the assay in peat‐based compost is consistent with that in sand. At an inoculum concentration of 10 CFU g^−1^, an average of 16 gene copies was detected. At 10^6^ CFU g^−1^, an average of 3.7*10^5^ was detected, indicating reduced detection at higher inoculum concentrations (Fig. 5).

**Figure 6:**
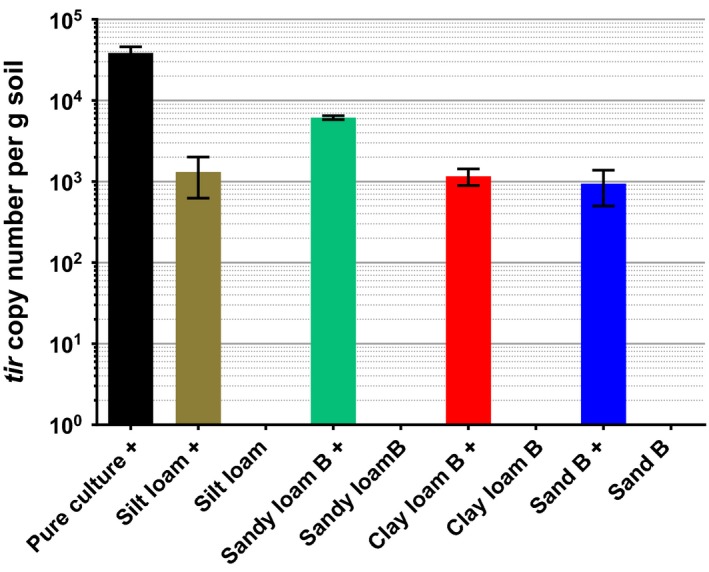
Pristine soil samples in which no *tir* gene copies were detected. + indicates samples inoculated with 10^5^ CFU *E*.* coli* O157 per g. Pure culture indicates inoculated water without any soil, as a positive control. Each condition was tested in duplicate. Error bars indicate SEM.

**Figure 7:**
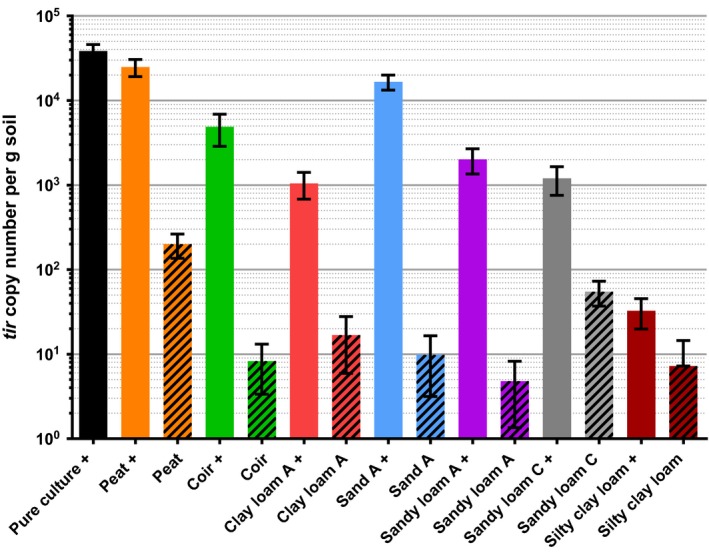
*tir* copy numbers detected in a range of pristine soil and growing media samples, compared with samples inoculated with 10^5^ CFU *E*.* coli* O157 per g. + indicates samples inoculated with the bacteria. Pure culture indicates inoculated water without any soil, as a positive control. Each condition was tested in duplicate. Error bars indicate SEM.

The assay was tested on nine soil samples of agricultural origin, in addition to peat and coir composts. Each sample was tested with and without an inoculation of 10^5^ CFU *E*.* coli* O157 per g soil. A positive control was generated by inoculating the same concentration of bacteria into sterile water, and a negative control was produced by using pure sterile water. For each sample, inoculating with bacteria increased detection of *tir* copy number, however each inoculated sample was found to be statistically lower than the positive control (Figs 6 and 7). Uninoculated peat‐based compost contained 200 *tir* copies per gram, while sandy loam C contained 55 copies per gram and clay loam A contained 17 copies per gram. The negative control contained no *tir* gene copies.

Using the Pulsifier and filtration to concentrate large volumes of soil allows for the assessment of larger soil samples than previously possible, bypassing the need for a culture‐based pre‐enrichment step while allowing for the detection of VBNC pathogens, and reducing sample preparation time to < 2 h.

## Discussion

### Sample separation by pulsification

The Pulsifier proved to be far more effective than the Stomacher at facilitating the detection of *E*.* coli* O157 by qPCR (Fig. 1). Previous studies have shown that in food samples, there is not a significant difference in bacterial recovery between the two processors (Sharpe *et al*., 2000; Kang *et al*., 2001). However, both reports corroborate in describing the reduced debris produced by the Pulsifier. In this study, the structural integrity of the assessed soil matrices coupled with the reliance on filtration for sample separation and concentration affirm the need for minimal debris. It is likely that the reduction in *tir* gene detection observed from using the Stomacher is caused by excessive soil particulate forming a physical barrier over the filter membranes, preventing bacterial elution. Therefore, we recommend that the Pulsifier is used over the Stomacher in the assessment of complex environmental matrices.

### Sensitivity of detection

The method developed is capable of detecting *E*.* coli* O157 in peat‐based compost and in sand, sensitive to 10 CFU g^−1^. It can also detect the pathogen in a range of other growing media and soil of different compositions. The difference in sensitivity across soil types could be due to the different rates at which they block the 5 μm pore filter membrane, the step at which most sample loss occurs. This had been explored during the method development, and to optimize sample elution for different soil types, the use of filter membranes with greater pore sizes could be implemented. Replacing the filter membranes more frequently increases the quantity of sample retrieved following the filtration steps, but at the cost of more rapidly using resources.

There is evidence for loss of bacterial sample to filter membranes described in Figs 6 and 7; in inoculated soils, fewer *tir* gene copies were detected than in the positive control, which contained no soil. This reduction in *tir* genes could have been caused by the build‐up of soil on the filter membrane during the filtration steps, preventing the elution of the complete bacterial sample.

In this way, the importance of protocol optimization is emphasized. The peat sample allowed for the detection of *tir* genes closest to the concentration of the positive control, at 64.6%. As the method was developed and optimized using peat‐based compost, it could be expected that a higher percentage of the gene would be detected in peat samples than in other soil types. The method developed would have to undergo further optimization to increase its sensitivity for soil of different physicochemistry. While there is the potential for this assay to be attuned to other soil types, it could also be adjusted to different pathogens. The specificity of the method is dependent entirely on the primer used in the qPCR assay; therefore, any pathogen present in soil could theoretically be detected by using this assay.

The reduction in sensitivity of *tir* gene detection at high inoculum concentrations could be due to saturation of DNA molecules during the DNA isolation process, or in the qPCR reaction wells. This is not necessarily detrimental to the sensitivity of the assay, one North American study ranging across 50 cattle herds found that the upper limit of *E*.* coli* O157 shedding was at a concentration of 10^5^ CFU g^−1^ faeces (Zhao *et al*., 1995). Contamination of this magnitude can easily be detected by the assay, although greater accuracy may be achieved with lower concentrations of the pathogen.

### *tir* gene detection in pristine soil

The reason for the discrepancy in the correlation between *tir* copy number and CFU inoculated into the soil sample could be that there is a background level of *tir* genes in bacteria present in soil. There is evidence for this in that agricultural soil samples tested show copy numbers up to 200 g^−1^ in peat (Fig. 7), despite having no bacteria inoculated into the sample. Using media selective for *E*.* coli* O157, traditional culture methods found the pristine peat compost negative for the pathogen. This limits the origin of the detected *tir* genes to either dead cells or those in a VBNC state.

Different quantities of the gene were also detected across pristine samples of different soil types, for example none were detected in silt loam, and eight copies per gram were found in coir compost (Fig. 7). It is also worth mentioning that as the silty clay loam positive control samples detected only a fraction of the *tir* genes inoculated into them (Fig. 7), the quantity detected in their corresponding pristine samples may also be a fraction of the true copy number of *tir* genes they harbour.

The number of *tir* gene copies detected in pristine soils differed across soil samples of similar compositions but from different locations, for example sandy loam A and C (Fig. 7). This suggests that contamination of agricultural soils by *E*.* coli* O157 may depend more on circumstances specific to each site, than an endemic contamination of soils by the pathogen favouring certain soil compositions. Optimization of the assay for different soil types will help to provide a more accurate reflection of the presence of endemic pathogenic genes across a range of sites.

Considering the sources of contamination, the existence of *tir*‐positive *E*.* coli* O157 in soils across disparate agricultural sites is plausible. Birds are known to shed the pathogen (Wallace *et al*., 1997; Chandran and Mazumder, 2014), one study mentioning a potentially large population of infected gulls roosting near to farmland (Wallace *et al*., 1997). There is also the possibility of the land being fertilized with contaminated manure or slurry, where cattle have been known to shed > 10^4^ CFU *E*.* coli* O157:H7 per g faeces (Matthews *et al*., 2006). This study has detected the *tir* gene in seven of the eleven soil samples tested; however, a range of factors may affect their detection by the described qPCR assay. PCR inhibitors or continued adhesion to soil particles of certain types could possibly impede the detection of the gene.

Preliminary work carried out in this study determined that the sterilization procedure of autoclaving soil for 30 min at 123 °C could reduce gene copy number by 5 orders of magnitude; however, this procedure was unable to destroy the DNA entirely. In addition to heat sterilization, soil samples were also sonicated and treated with deoxyribonuclease I (Sigma), although this failed to further reduce the *tir* gene signal. This could be due to protection of DNA molecules from degradation by adsorption to soil particles. This protection has been observed in clay particles, where DNase I treatment failed to completely degrade DNA molecules in the presence of clay minerals (Demaneche *et al*., 2001).

We advise that if used to screen soil samples for contamination, this assay should use sterile soil as a negative control. As neither the culture data nor the qPCR assay eliminate the possibility that the *tir* gene copies detected in pristine peat‐based compost originate in VBNC *E*.* coli* O157 cells, any positive result in agricutural soil should be investigated further as a potential threat to food safety. By screening a greater number of soil samples and subsequent investigation, we will have a better understanding of the food safety implications of a positive result generated by this assay and the cause of their apparent ubiquity.

With minor alterations, this method could be utilized to detect and monitor a range of pathogens contaminating a range of soil types. Future research could work to optimize the protocol for different soil compositions or even plant matter, to provide a postharvest screen for contamination of fresh produce. At present, the procedure offers a rapid, quantitative risk assessment of the presence of *E*.* coli* O157 in soils and growing media, with applications in the diagnostics of occurring outbreaks for source identification and the prevention of those that may otherwise go undetected.

## Experimental procedures

### Bacteria and soil preparation

The strain used was *E*.* coli* O157:H7 NCTC 12900. Peat‐based compost was used in the initial method development, purchased from a commercial supplier (Wickes, Northampton, UK). Coir compost was supplied by Vitacress Salads ltd (Dr Graham Clarkson); agricultural soil samples from sites around the United Kingdom were kindly supplied by ADAS (Dr Lizzie Sagoo).

Soil and growing media were sterilized prior to inoculation with bacteria by thinly spreading each sample inside a loosely closed bag and autoclaving in a Priorclave benchtop autoclave at 123 °C for 30 min. The testing of agricultural samples was carried out without sterilization.

### Bacterial growth and soil inoculation

*E*.* coli* O157 was grown in brain heart infusion broth (Oxoid, Basingstoke, UK) overnight at 37 °C to stationary phase. Bacterial suspensions of different concentrations were produced by diluting in PBS (Oxoid, Basingstoke, UK).

Soil samples (25 g) were added to separate BagFilter P Stomacher bags (Interscience). One millilitre of each suspension was added to each soil sample and left to adhere to the sample for 5 min; 225 ml ddH_2_O was then added to each bag.

To determine the presence of viable *E*.* coli* O157 in soil samples, pristine soil samples were prepared as described above, and 100 μl of pulsified sample was added to Petri dishes containing selective medium CHROMagar O157 (Invitrogen). The agar plates were incubated at 37 °C overnight.

### DNase treatment of soil

Soil samples (200 g) were autoclaved at 123 °C for 30 min, and then added to 400 ml water with 5 mM Ca^2+^ and 10 μg ml^−1^ DNase I (Sigma‐Aldrich). Samples were incubated for 1 h at 37 °C with gentle agitation. Soil samples were drained of water and autoclaved again under the same conditions as before, prior to inoculation.

### Sample separation from soil matrix

The bagged sample was pulsified in a Pulsifier™ (Microgen Bioproducts ltd., Camberley, UK) for 30 s, then the suspension was aspirated through the BagFilter P bag 250 μm pore filter, so that larger soil particles were excluded from downstream processes. To test the Stomacher, bagged samples were homogenized for 2 min using a Stomacher 400 (Tekmar, Newton Aycliffe, UK). Aliquots of the suspension (25 ml) were added to a filtration unit fitted with a 47 mm diameter PVDF filter membrane with a pore size of 5 μm (Millipore, Watford, UK). Using vacuum filtration, the suspension was filtered through into a receiver beneath. After the completion of filtration of the first 25 ml, another 25 ml was added. The membrane was renewed for each 50 ml that had been filtered; 200 ml of the suspension in total for each sample was filtered through to the receiver.

The filtrate underwent a second round of vacuum filtration, using a 47 mm diameter mixed cellulose–ester membrane with a pore size of 0.22 μm (Millipore). Once the entirety of the sample had filtered through, the filter membrane was removed and added to a 15 ml tube containing 1 ml PBS. The tube was shaken using a vortex mixer for 2 min, in the manner previously described by Wolffs *et al*. (Wolffs *et al*., 2006); then the membrane was removed.

### DNA extraction and qPCR assay

The 1 ml suspension was transferred to a 1.5 ml Eppendorf tube and centrifuged at 10 000 × g for 5 min. The supernatant was removed, and the pellet was resuspended in 100 μl in PBS for DNA extraction using the Powersoil DNA Isolation kit (Mo Bio) according to the manufacturer's instructions. DNA samples were stored at −20 °C until use.

qPCR was carried out according to the Genesig *E*.* coli* O157:H7 kit instructions, targeting the *tir* gene specific to *E*.* coli* O157. The qPCR method consisted of a 10 min warming stage at 95 °C and then 50 cycles of a 10 s step at 95 °C, followed by a 60 s step at 60 °C (Primer Design, Southampton, UK). Amplification was carried out using a Bio‐Rad iQ5 cycler. Each sample was measured with at least three replicate wells.

### Data analyses

Statistical difference between qPCR data sets was calculated using one‐way analysis of variance and Fisher's least significant difference test.

To calculate *tir* gene copy number per gram of soil, each qPCR plate contained a standard curve containing known concentrations of the gene. The cT values for test samples were compared against the standard curve, giving *tir* copy number per microlitre of DNA sample. These values were multiplied to correspond to the quantity of DNA sample per gram of soil.

## Conflict of interest

None declared.
